# A Study on the Role of Affective Feedback in Robot-Assisted Learning

**DOI:** 10.3390/s23031181

**Published:** 2023-01-20

**Authors:** Gabriela Błażejowska, Łukasz Gruba, Bipin Indurkhya, Artur Gunia

**Affiliations:** 1 Nextbank Software, 30-085 Krakow, Poland; 2 Kitopi, 30-383 Krakow, Poland; 3 Cognitive Science Department, Institute of Philosophy, Jagiellonian University, 31-007 Krakow, Poland

**Keywords:** human–robot interaction, programming education, social robots, Miro-E, emotion recognition, affective computing

## Abstract

In recent years, there have been many approaches to using robots to teach computer programming. In intelligent tutoring systems and computer-aided learning, there is also some research to show that affective feedback to the student increases learning efficiency. However, a few studies on the role of incorporating an emotional personality in the robot in robot-assisted learning have found different results. To explore this issue further, we conducted a pilot study to investigate the effect of positive verbal encouragement and non-verbal emotive behaviour of the Miro-E robot during a robot-assisted programming session. The participants were tasked to program the robot’s behaviour. In the experimental group, the robot monitored the participants’ emotional state via their facial expressions, and provided affective feedback to the participants after completing each task. In the control group, the robot responded in a neutral way. The participants filled out a questionnaire before and after the programming session. The results show a positive reaction of the participants to the robot and the exercise. Though the number of participants was small, as the experiment was conducted during the pandemic, a qualitative analysis of the data was carried out. We found that the greatest affective outcome of the session was for students who had little experience or interest in programming before. We also found that the affective expressions of the robot had a negative impact on its likeability, revealing vestiges of the uncanny valley effect.

## 1. Introduction

There is a long history of using robots to teach computer programming to children and college students [1,2,3,4,5]. A robot is a tangible, physical device that can be programmed to make different movements, and display different behaviours. This makes robots more interesting to novice programmers compared to writing “Hello World” on a display.

In using a robot as a vehicle to teach programming, one critical factor is what kind of personality should be given to the robot to make it more effective. Previous research on intelligent tutoring systems has demonstrated that an affective interface yields better learning outcomes [6]. However, for a robot tutor, the results are mixed. Some studies have found that endowing a robot with an emotional personality is effective [7,8], but others were not able to find any significant effect [9,10].

To explore this issue further, we conducted a pilot study where a dog-like robot (Miro-E) was used to teach programming to children (11–15 yrs) under two different conditions: a neutral-personality condition and an emotional-feedback condition. In the emotional-feedback condition, the robot sensed the emotional state of the students through their facial expressions, and gave encouragement through verbal and non-verbal modalities. The verbal feedback took the form of praising the student, and non-verbal feedback included wagging the tail, moving the head, and wiggling the ears. Throughout the experiment, we monitored the emotional state of the students through their facial expressions.

This study was conducted during the pandemic, so the number of participants was small. However, we carried out a qualitative analysis of our observations, which is reported here.

This paper is organised as follows. In Section 2 we review the related research. Section 3 presents our experimental design, followed by the details of the experiment in Section 4. The results and discussion are presented in Section 5 and Section 6, respectively, followed by the conclusions in Section 7.

## 2. Related Work

### 2.1. Robots in Education

Over the years, there have been many attempts at using robots for educational purposes. For example, in an older study [7], a robot was used to teach an artificial language to primary-school students. The robot was designed to offer two levels of social behaviours—neutral and supportive. Participants who studied with the supportive robot achieved significantly higher results and reported higher motivation levels.

In an earlier survey [3], it was observed that 74% of the reviewed studies found support for robots as an effective teaching tool. A later survey [5] reported that the introduction of robotics in the curriculum increases children’s interest in engineering, and allows children to engage in interactive and engaging learning experiences.

Sharma et al. [11], while studying how collaboration and engagement affect children’s attitudes towards programming, asked the children to manipulate digital robots (avatars) as a priming activity before starting programming exercises. Van den Berghe et al. [12] directly compared how robots as opposed to avatars affected children’s cooperation while learning programming, and found robots to be more effective than avatars.

According to a meta-analysis of studies on the efficacy of social robots in education [13], robot tutors are not at the same level as human tutors: students show lower learning outcomes when directly comparing studying with a robot versus a human tutor. However, there are some benefits of robots over humans in education. It is more economically viable to provide devices to each student than one-on-one tutoring with a human teacher. Technology also allows the curriculum to be customised to the learning pace of each student.

Robots have an advantage over screen-based educational applications, because they increase cognitive learning gains [14] and elicit more social engagement from students [15], compared to screen educational content. The use of robots also appears to be effective for interactive courses where technology is the subject of the course. In this case, the robots engage students in critical and computational thinking, problem solving, and collaboration [16,17,18,19]. Moreover, the use of robots is motivating for both the student and the teacher designing the course [20,21].

In particular, when it comes to teaching programming, physical devices have an advantage that the student can see the effect of executing an algorithm. Devices such as micro:bit [22] and robots [19,23] have been found to be effective.

As robots do not yet have the capability to be general all-round teachers and perform better than humans, many studies choose to compare two different robot behaviours with each other, instead of measuring one robot behaviour against a human tutor. For example, one could compare a socially supportive behaviour that engages in a social dialogue with a neutral behaviour that focuses on a plain knowledge transfer [7]. Or, one could compare the tutor condition—a robot that is focused on guiding a learner in solving increasingly complex problems in a scaffolding fashion—with the peer-like behaviour to support engagement [7,8]. This is the approach adapted in our study.

### 2.2. The Role of Affective Feedback

Affective feedback is known to have a major impact when a human is the teacher or the trainer [24,25]. In computer-based tutoring systems, affective feedback is also found to be effective [6].

In human–robot interaction, however, incorporating an emotional personality into the robot has yielded mixed results. For instance, Saerbeck et al. [7] found that incorporating a life-like social personality in a virtual actor increases the learning efficiency of students. Zaga et al. [8] compared the effect of two different social personalities of a robot—a peer and a tutor—and found the peer personality to be more effective. However, Konijn & Hoorn [10] used the humanoid robot NAO to teach primary-school children the multiplication table. Their study compared a robot using neutral language (providing feedback with only variations of ‘correct’ and ‘incorrect’) to a more social and encouraging robot. Techniques used to create social interaction included addressing the participant by name, having the robot follow their gaze, and using encouraging gestures and language (such as ’well done’, ’fantastic’). The results showed no significant difference between the two groups when comparing across all participants. Students with below-average test scores performed worse with social robot tutoring than with a neutral robot. Similarly, another study [9] did not find benefits of social behaviours of the robot for a lesson on prime numbers.

One reason for this effect might be that social behaviours from a robot can distract from the lesson and increase cognitive load. It could also be that students are surprised or unsettled by robots showing such behaviours. Studies showing a lack of benefit from social behaviours in a robot [10,26] have compared the cognitive outcomes, measuring differences in test scores. In our study, we chose to focus on qualitative feedback from the participants to assess the effect of affective feedback on learning.

### 2.3. Emotion Recognition

Knowing the emotional state of a student is important from the point of view of teaching. Having the ability to recognise if a student is bored, frustrated, excited, or in any other emotional state is a valuable skill for every teacher. For example, if a student is bored, it could be an indicator that they have lost focus or that they may already be familiar with a particular topic and are ready to move on [27]. Another example could be when a student is frustrated, which most probably means that they are experiencing some difficulties with the learning material.

There are several aspects of emotions and many available techniques for measuring them [28]. One key issue is the dimensions of emotions, and the literature [29] provides the following list: (1) arousal—whether an emotion turns on, activates an action, or inhibits it; (2) value—whether an emotion has positive or negative value for a person; (3) intensity—whether the strength with which the emotion is perceived is low or high; (4) duration—time duration of a given emotion; (5) frequency of occurrence—how often does a given emotion occur; (6) time dimension—whether the emotion is retrospective (e.g., relief), real (e.g., pleasure), or prospective (e.g., hope). Another factor is the set of basic emotions in terms of which all other emotions can be expressed. The Plutchik model [30] provides one such set of basic emotions.

Tools for measuring emotions can be divided into three groups: (1) psychological, mainly subjective, and retrospective reporting of one’s own emotional states (e.g., via verbal reports, questionnaires); (2) physiological objective tests that measure physiological responses using sensors (e.g., electrocardiogram (ECG), electroencephalogram (EEG), galvanic skin response (GSR)); and (3) behavioural objective measures based on bodily manifestations (e.g., facial expression, voice prosody, body posture) [31].

A commonly used emotion recognition technique is based on the set of emotions proposed by Paul Ekman [32,33], and it has been used successfully in educational tests [34,35]. It is a discrete model with six basic emotions: anger, disgust, fear, happiness, sadness, and surprise. Later on, the list was expanded to include emotions of contempt, guilt, embarrassment, relief, or satisfaction. However, the original model is often used, especially for automated emotion recognition [36], as it is based on a relatively small set of well-defined and easily distinguishable states.

The Ekman model can be used in automated techniques for detecting emotions, which in practice consist of detecting emotions from changes in a facial micro-expression (a facial expression that only lasts for a short moment). Detecting emotion from a micro-expression is not without its drawbacks, such as the ability to assess only basic emotions or the fact that it does not always work for all respondents. However, thanks to information technology (IT) solutions, it is a quick and relatively simple method used to evaluate emotions in changing conditions. Though emotions can be recognised from facial images using automated techniques, some sort of an image and pattern recognition algorithm has to be involved. Creating any image recognition algorithm manually can be difficult and error prone. In recent years, the most popular approach to this is to use machine learning [37,38,39].

Dimensional models present a different approach to classifying emotions. As opposed to discrete models, where emotions are defined as distinct states, in dimensional models all emotional states are described by two or more dimensions. Thus, emotional states form a spectrum rather than separate groups. The dimensions used to describe emotions are usually based on intensity and whether the emotion is positive or negative. One of the dimensional models was proposed by James Russell [40]. It is known as the circumplex model of emotions [40]. It is a two-dimensional model where the dimensions are valence (whether the emotion is positive or negative) and arousal (the level of activation, e.g., calm vs. excited). Placing valence on the horizontal axis and arousal on the vertical axis, all emotions are placed in the circular space defined by these two dimensions [41]. This is the model used in our study.

## 3. Study Design

The main objective of this research was to study how the emotional response of the robot affects the learning process and the emotional attitude of the student. More specifically, our aim was to address whether the process of learning can be more effective when assisted by an AI that can engage emotionally with the student by managing the difficulty of the task based on the emotional feedback and by providing encouraging verbal feedback. Consequently, the hypothesis of our study was that participants who receive encouragement and emotional support from an empathetic robot will be more engaged in the lessons, will be less frustrated by failures, and will have a higher interest in continuing their development in the field of computer science.

To validate this hypothesis, we conducted a study where children (11–15 yrs) were asked to complete the task of programming a robot while interacting with it. A Miro-E robot was used in the study; this is described below.

### 3.1. The Miro-E Robot

**Figure 1:**
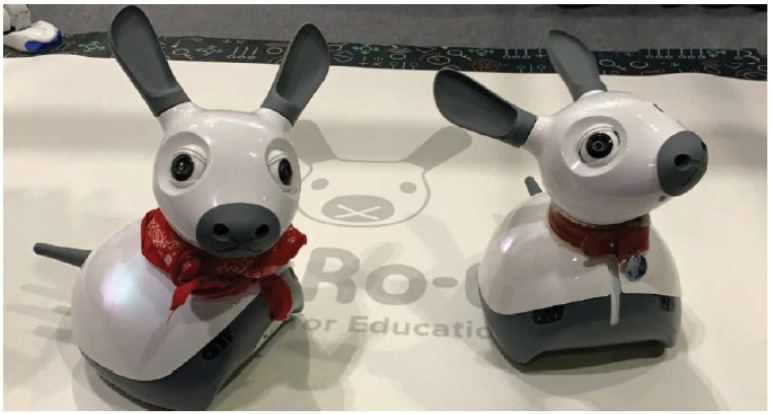
Miro-E robot.

Miro-E is a small robot developed by Consequential Robotics (Figure 1), and has animal-like features. It is designed to look like a hybrid of different pet animals. It has an articulated head with ears and eyes, haptic sensors in the head and the body that react to touch, and a microphone to detect sound. The eyes, ears, tail, and head can be moved to express affective states. The robot’s behaviour can be programmed using a block interface based on the Blockly library. By combining sensor readings with conditional logic, one can create programs so that the robot reacts when touched or when it hears a clap. The programming interface also allows for running separate scripts on the robot in parallel: this feature is used so that the robot can run programs written by the participants during the study while running reaction scripts to provide affective feedback at the same time.

The reaction scripts were written in Python (URL: https://www.python.org/ accessed on 4 January 2023), using the Rospy library (URL: http://wiki.ros.org/rospy accessed on 4 January 2023) and the Miro Interface modules to control the hardware. The script files were uploaded to the robot’s memory, and were executed on demand by running them with a Python interpreter from the command line via secure shell protocol (SSH).

**Figure 2:**
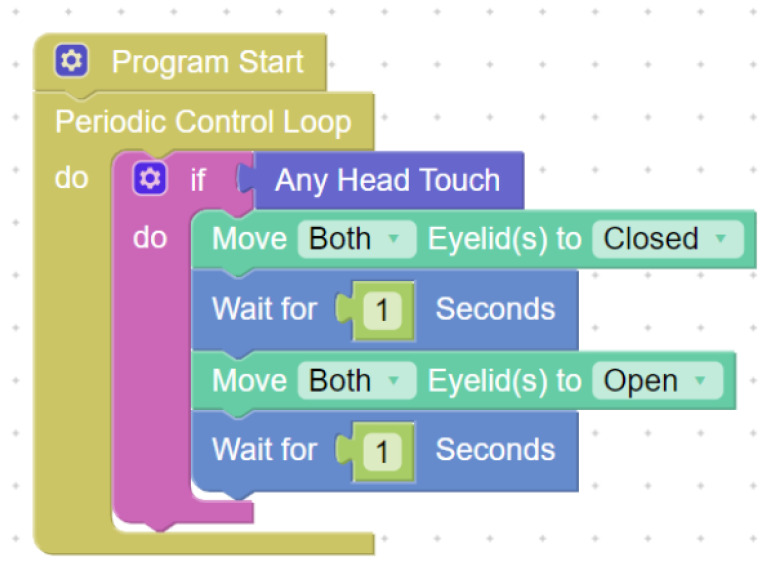
An example program written in MiroCode’s visual interface.

The study participants used MiroCode (URL: https://www.miro-e.com/mirocode accessed on 4 January 2023) (Figure 2) to program the robot. MiroCode is a visual interface that uses a block representation of the robot’s actions. A program is created by chaining together blocks that describe sequential actions of the robot. This web application was created by Consequential Robotics specifically for teaching programming using Google’s open-source library Blockly (URL: https://developers.google.com/blockly accessed on 4 January 2023).

Robot capabilities are divided into separate modules grouped by themes (motion, time sequence, sensors, etc.). The programming interface is friendly to beginners as it requires little knowledge of syntax, and each block explains the action taken by the robot. The blocks can be executed in a sequence or in a loop. The cloud version of MiroCode, MiroCloud, was used in this study.

### 3.2. Emotion Recognition Module

**Figure 3:**
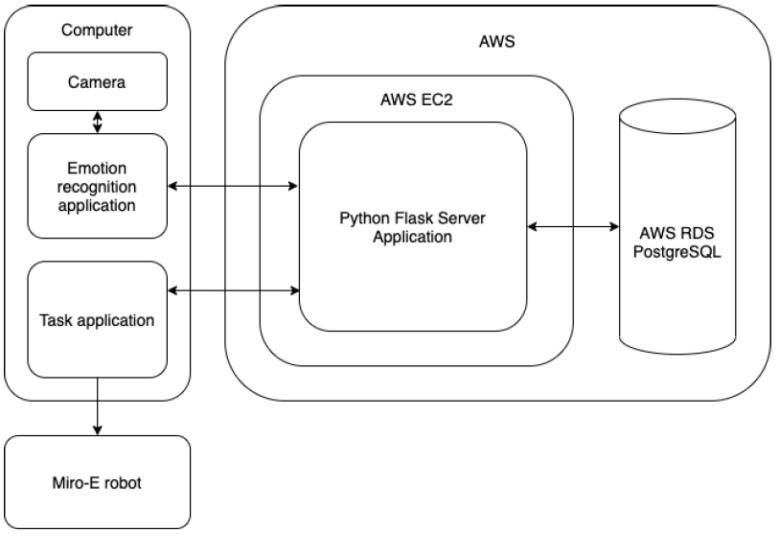
Architecture diagram. Please refer to the text for an explanation of the acronyms.

The emotion recognition module implemented for this study was divided into two modules running in parallel. The first module was a standalone client-side application that is responsible for collecting facial images from the laptop camera and generating their valence and arousal values. The second module was a server-side application responsible for storage, managing the collected data, and computing end results. The architecture is shown in Figure 3.

The client-side module ran on the open-source software library Keras (Keras library accessed on 15 December 2022; URL: https://keras.io/about/) with trained models for valence and arousal. The video was captured from the laptop camera and the frames were continuously fed into the model, which returned the valence and arousal values for each frame. These values were sent to the server-side application via the http protocol. It prepared the records and saved them in the database for further calculations. The server-side application ran on Amazon Web Services (AWS) (cloud computing with AWS accessed on 15 December 2022; URL: https://aws.amazon.com/what-is-aws/). A single Amazon Elastic Compute Cloud (EC2) (Amazon EC2 accessed on 15 December 2022; URL: https://aws.amazon.com/ec2/) instance served as the host for a Python application implemented in the Flask (Flask accessed on 15 December 2022; URL: https://palletsprojects.com/p/flask/) framework. It exposed a set of REST services (What is REST, accessed on 15 December 2022; URL: https://restfulapi.net/) which were being called by the client-side application (emotion recognition application). An instance of the AWS Relational Database Service (RDS) (Amazon Relational Database Service (RDS) accessed on 15 December 2022; URL: https://aws.amazon.com/rds/) was hosted in the cloud, and was used to store all the data collected through the experiment in the open-source database system PostgreSQL (URL: https://www.postgresql.org/ accessed on 15 December 2022).

The server-side application also returned the results for a given time-frame. Timestamps were taken when a student started and ended solving a given task. These were attached to the request sent to the server-side application, which retrieved all the data during this time period to compute the end result according to the algorithm explained in Section 3.2.2.

#### 3.2.1. Training the Emotion Recognition Model

For recognising emotions from facial images, a machine learning model was trained using AffectNet, which is currently the largest facial expression data set, with each image annotated with a categorical label, and its valence and arousal values based on the circumplex model [42].

The AffectNet data set contains more than one million images, of which 440,000 were annotated manually, and the rest were annotated automatically. We observed that these images are distributed unevenly across the valence and arousal spectrum: most of the images covered a small range of valence and arousal values, and there were few images with very high or very low valence and arousal values. Such an uneven distribution of data is not ideal for training.

To address this problem, we divided the entire range of values (from −1.0 to 1.0) into small intervals (in steps of 0.01, so −1.00 to −0.99, −0.99 to −0.98, and so on) and considered how many images fell in each interval. With trial and error, we found that by taking at most 400 images from each interval (when an interval had less than 400 images, we took all of them), we could create a more uniform distribution across the entire spectrum, and still have a large enough dataset to train the model. This procedure was performed once across the valence spectrum and once across the arousal spectrum.

To address the problem that the images were of different resolutions, we scaled all the images down to 200 × 200 pixels. As the Xception network is designed for images of size 299 × 299 pixels, the first input layer had to be readjusted to work with different image sizes. As a separate data set had to be used for training the valence model and the arousal model, all the operations mentioned before had to be repeated twice, once for each model. This resulted in two final data sets that could be used during training, one for valence and the other for arousal.

A random split was performed to divide the data sets into training and validation categories: 80% were assigned to the training data set and the remaining 20% to the validation data set.

The parameter values used for the network were as follows. The network used was an Xception pre-trained on the ImageNet data set (ImageNet accessed on 15 December 2022; URL: https://www.image-net.org/). The batch size was set to 32 images. The loss function used during training was mean absolute error (MAE) [43]. The Paperspace (About Paperspace Gradient, accessed on 15 December 2022; URL: https://docs.paperspace.com/gradient/) platform was used to provide more computing capacity. Training occurred on a single Free GPU + instance (Instance Types available in the Free Tier, accessed on 15 December 2022; URL: https://docs.paperspace.com/gradient/more/instance-types/free-instances#instancetypes-available-in-the-free-tier) equipped with 8 CPUs, 30 GB RAM, and a Quadro M4000 GPU. The value loss achieved after training was 0.244 for the valence model and 0.258 for the arousal model. Considering that the values for both parameters have a range from −1.0 to 1.0, this translates into a 12.2% error rate for the valence model and 12.9% for the arousal model.

#### 3.2.2. Emotion Computing Algorithm

An algorithm was created to aggregate valence and arousal values over time into one of the three categories, positive, negative, or neutral. These aggregated values were used by the experiment control system to control the task flow.

Only measurements taken while solving a particular task were considered. We assumed that the participants would have a neutral facial expression most of the time. A high-pass filter was used to filter out measurements of low significance. Euclidean distance was used for filtering as follows:

(1) $$\sqrt{valence^{2}\times arousal^{2}}\geq 0.3$$

Different threshold values were tried, and in the end, 0.3 was chosen as a good compromise between filtering out noise and not filtering too much. This allowed most of the unimportant measurements to be filtered out.

Weights were used to give more significance to the measurements taken at the end of the task, as they would be related to the participant finding the final solution. A hyperbolic tangent function was used to calculate the weights as follows:

(2) weight(t)=tanh(rel_time(t)×π)

where rel_time is a function to compute the relative time of the measurement compared to the entire duration of the task execution. The relative time was calculated using the formula:

(3) $$rel\_time\left(t\right)=\frac{t-t_{min}}{t_{max}-t_{min}}$$

where $t_{min}$ and $t_{max}$ are the start and end times of task execution.

After computing the weights for every measurement taken for a particular task, the final result was computed as follows.

(4) $$result=\frac{\sum_{i=1}^{m\_size}m_{i}\times weight\left(t_{i}\right)}{\sum_{j=1}^{m\_size}weight\left(t_{j}\right)}$$

where m_size is the size of the measurement set, $m_{i}$ is a particular measurement value, and $t_{i}$ is the measurement time.

**Figure 4:**
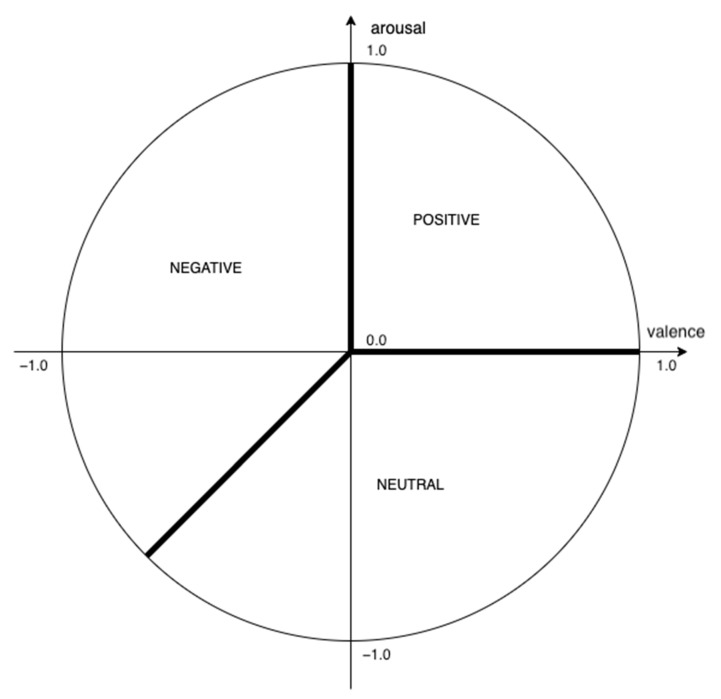
Visualisation of the valence-arousal plane for the final result calculation.

This formula was applied to the valence and arousal measurements separately, and the calculated values were used to determine the final outcome depending on where the results fell in the circumplex model. A visualisation of this is shown in Figure 4.

### 3.3. Experimental Set-Up

The main activity in this study was for the participants to solve programming tasks to control the Miro-E robot in a MiroCode environment. While they were engaged in this task, their emotional states were analysed from their facial images (taken by the laptop camera). Based on these emotional states, the robot provided appropriate affective feedback in both verbal and non-verbal modalities. The feedback could be praising the participant for completing a task successfully, or congratulating them on finishing a tricky task. Non-verbal feedback included wagging the tail, moving the head, and wiggling the ears. The robot then presented the next task to the participant.

The system also decided whether the participant should skip some tasks. If the participant completed the current task in a short time (less than the preset threshold), and the emotion recognition module found that the participant had a positive reaction, then the system would skip over the next task.

For the control group, the Miro-E robot was only a vehicle for the programming tasks (it only performed the actions programmed by the participant): it took no actions of its own. The robot announced the next task in a neutral manner: ’Start Task 3’.

All participants were also asked to fill out a pre-test questionnaire to assess their previous programming experience, and a post-test questionnaire to gather information about their experience during the study. Data collected from the questionnaires were used to determine the impact of affective feedback provided to participants in the experimental group.

## 4. Experiment

### 4.1. Participants

The experiment was conducted in the period June–July 2021. Because of the COVID-19 pandemic, it was difficult to find participants, but we managed to recruit nine participants (3F, 6M; 11–15 yrs) to take part in the activity of learning to program a robot. All participants had sufficient English knowledge to allow them to use the robot’s interface. English proficiency was determined from the participant’s self-declaration—the call for participation mentioned that English would be required. Moreover, all the participants had attended several years of primary school with mandatory English lessons. Four participants were placed in the control group and five in the experimental group.

### 4.2. Coding Tasks

The participants were given the task of writing programs to control the Miro-E Robot. A set of ten programming tasks were prepared, which were expected to take about 40 min to complete. The tasks were progressively more difficult, with later tasks building on earlier tasks, and with each section of the worksheet introducing new concepts or block modules.

The first task in each section was to open an example program that had been saved on the laptop, read the code, and explain what it does to the researcher. Then, the participant was asked to run the program on the robot and observe if their predictions were correct. This familiarised the participant with what the blocks in the program did, and provided context for the next task.

The second task in each section was a coding exercise. The participant was given a desired behaviour of the robot (for example, to make Miro-E walk in a square), and was asked to write a program to make Miro-E behave in that way. The participants were asked to do this independently: the researcher helped (if needed) only with language difficulties.

The final section, *Functions*, did not have a given outcome of the task, but introduced the concept of functions as reusable blocks of code that are defined once, but then they can be called from different places in the program. The example code contained three function slots that participants could complete as they wanted using the knowledge gained from the previous sections. The objective of this task was for the participants to understand how a function code is triggered from the main program.

### 4.3. Procedure

Informed consent was obtained from the legal guardian of each participant after explaining to them the following aspects of the study:

- The video of facial expressions from the laptop camera is used for emotion-recognition.
- No identifying information of the participants is disclosed in the study.
- Data from the participants are used anonymously and in aggregate.

Before starting with the programming tasks, each participant was asked to fill out a pre-test questionnaire containing the following questions (items 3 and 4 used a five-point Likert scale):

1. Participant identification number.
2. Grade in school.
3. General interest in programming (1: ‘no interest’; 5: ‘great interest’).
4. Previous coding experience (1: ‘no experience’; 5: ‘much experience’).
5. Familiar programming environments.

Then, the participant was asked to sit at a large table with a laptop that showed the MiroCode interface, with the Miro-E robot on an adjacent table. Throughout the study, a researcher sat next to the participant to explain the experiment, help with language problems, and provide input to the system when starting or finishing a task.

The experiment was started with the researcher showing the participant the worksheet with tasks, and explaining the structure and the goal of each section. The participant was then given a tour of the MiroCode interface: where to find blocks, how to run the code on the robot, and how to open example programs. Participants were encouraged to explore solutions even when they were not sure about their correctness. Finally, the researcher told the participant to feel free to ask any questions about the language or meaning of certain blocks during the experiment.

The participant then started the first task. When the participant started a task, the researcher entered this into the system. The participant then completed the task, usually running several versions of their code on the Miro-E robot before succeeding. In tasks that involved moving the robot, the researcher positioned the robot next to the participant, or in a location where the robot could complete the movement without encountering an obstacle.

When the participant completed a task, the researcher entered the task-end response into the system using a mobile device.

This procedure was repeated until all the tasks were completed. The procedure was the same for both the experimental and the control groups, with the only difference being that (as explained above in Section 3.3) for the experimental group, Miro-E provided affective feedback, and the progression of tasks depended on the participant’s affective state while completing the tasks.

After the participant finished all the tasks, they were asked to complete the following post-test questionnaire (items (1)–(4) used a five-point Likert scale):

1. Rate Miro-E’s behaviour (1: ‘unpleasant/rude’; 5: ’pleasant/nice’).
2. Rate your enjoyment of the session (1: ‘didn’t like it at all’; 5: ’liked it a lot’).
3. Rate your general interest in programming (1: ‘no interest’; 5: ‘great interest’).
4. Would you be interested in another session with Miro? (1: ‘no interest’; 5: ‘great interest’).
5. Did any task make you feel frustrated?
6. If yes, which task(s)?
7. Did any task make you feel accomplished?
8. If yes, which task(s)?

## 5. Results

As this study was conducted during the COVID-19 pandemic, the groups were relatively small: four participants were in the control group and five were in the experimental group. Nonetheless, we analysed the affective outcomes: the feelings of the participants towards the Miro-E robot, towards the experiment, and towards programming in general. The results of the post-test questionnaire are summarised in Table 1.

All the participants finished all the given tasks—no one stopped the experiment before it ended. For one participant in the control group, all the data could not be recorded due to a technical difficulty, so this participant was excluded from the analysis. None of the participants reported feeling frustrated by any of the tasks.

Six participants (4 from the experimental group, 2 from the control group) reported that a task made them feel accomplished or happy. One participant from the experimental group reported that task one, moving the robot, affected her or him in this way. Five participants (3 from the experimental group, 2 from the control group) pointed to task 10 as making them feel accomplished.

**Figure 5:**
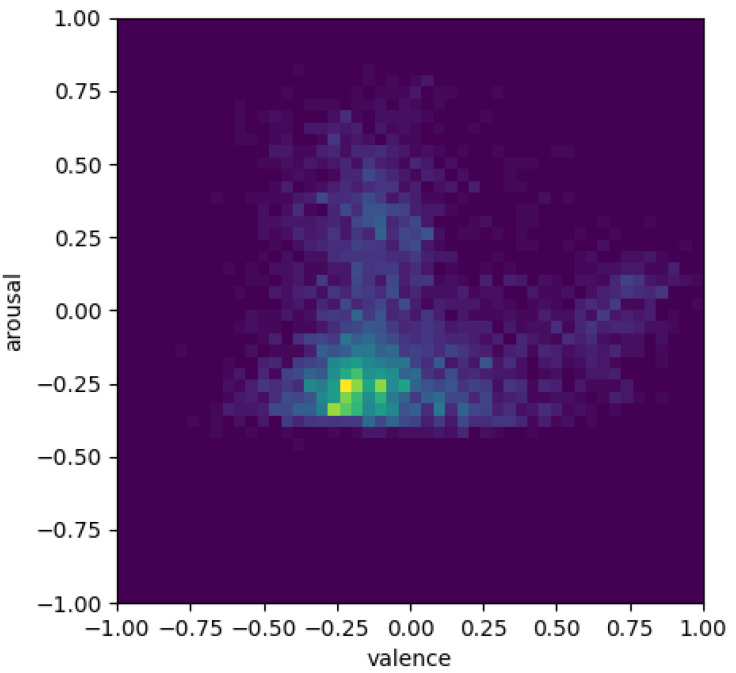
Heatmap showing the valence and arousal for the control group participants.

**Figure 6:**
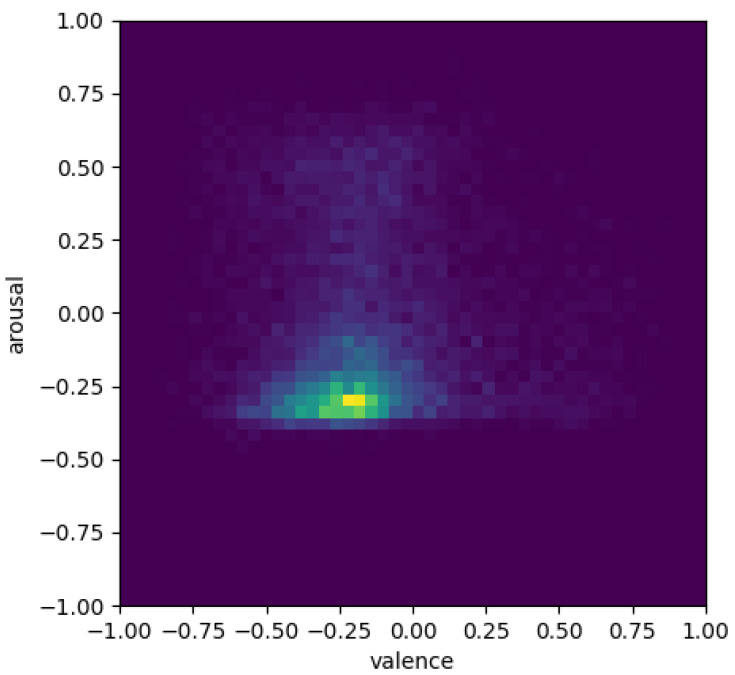
Heatmap showing the valence and arousal for the experimental group participants.

The aggregate (over the participants) of the data collected by the emotion recognition module is shown as heat maps in Figure 5 and Figure 6. These heat maps can be interpreted qualitatively by comparing a segment of the obtained valence/arousal predictions to the ground truth values. Therefore, the heat map illustrates, in the 2-D valence and arousal space, the histograms of the ground truth labels of the test set and the corresponding predictions of the trained model. We can see that the heat points are mostly in the middle because the measurements were mostly neutral or shifted towards negative valence and arousal.

## 6. Discussion

The results of the survey show that all the participants rated Miro-E’s behaviour as friendly: on a five-point Likert scale with a range from “unpleasant, unfriendly” to “nice, friendly”, the average response was 4.8. In the control group, where the Miro-E robot exhibited only neutral behaviour and language, all the participants responded with a 5. The average in the experimental group was 4.6 (with two participants rating Miro-E’s friendliness at a 4). This suggests that the robot’s attempts at friendliness had a negative impact on its likeability. One participant in particular seemed visibly surprised, and moved away from the robot when starting the script for completing a task. This could be due to the uncanny valley effect [44].

These responses show that the Miro-E robot is perceived as friendly by itself, even when no additional behaviour to support this is programmed. This is by the design of the manufacturer, as the robot is aimed at younger children, and looks like a pet animal. Moreover, the tasks the participants were performing made the robot appear more friendly. The participants themselves were in charge of programming the robot and used its emotive features in their programs—making the robot wag its tail and wink in response to being touched. This kind of social behaviour did not trigger the same surprise reaction, as it was expected and programmed by the participant.

Another question asked in the survey related to the participants’ enjoyment of the programming session and whether they would like to take part in another session with the Miro-E robot. The average for the control group was 4.25, and for the experimental group was 4.6. Thus, fewer participants from the experimental group expressed an interest in a future lesson with Miro-E. One reason for this could be that prior experience with programming was higher in the experimental group compared to the control group, and for participants having more prior experience with programming, the tasks seemed easy, so they were not so interested in another session with Miro-E.

The aggregated heat maps of valence and arousal for participants in the control and the experimental groups (Figure 5 and Figure 6) show that the participants in the control group experienced higher overall valence and arousal values, while the experimental group’s heat map is concentrated mostly in the neutral region.

This suggests that the control group experienced more positive emotions compared to the experimental group. However, this could also mean that emotion recognition based on computer vision and facial micro-expressions was not very effective. In future, we need to incorporate other measures that are indicative of attention and interest besides the emotional state of the user.

The results from the survey conflict with the results of the valence and arousal graphs. This may be due to the courtesy bias, as the participants were rating the study while the researchers were in the room. Moreover, it is hard to ascertain satisfaction with the robot-based learning session by just comparing the results of the pretest and post-test surveys, especially as the emotional state of the participants was changing during the session.

Nonetheless, these results can be explained as follows. The survey results show considerable interest and satisfaction with the robot-assisted learning. However, the emotional recognition based on facial expressions suggest that a robotic assistant does not trigger a strong emotional state. This can also be interpreted positively in that the robot assistant does not distract from the required task.

The affective outcome of the study was measured by asking the participants about their interest in programming before and after the session (on a five-point Likert scale) with the robot. This showed an overall increase of 0.34 for the entire group: the increase was 0.25 for the control group and 0.4 for the experimental group. It should be noted that most of the participants did not change their answer (from pretest to post-test), but the participants with a low pre-test interest in programming showed an increase in the post-test.

**Figure 7:**
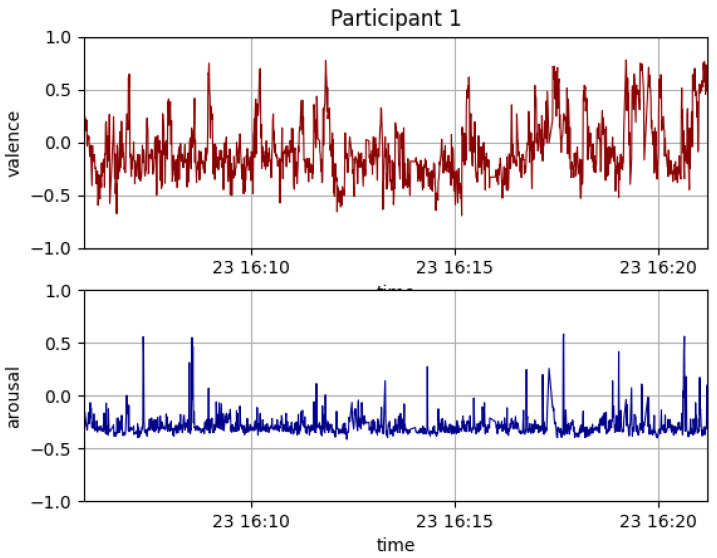
Graphs of valence and arousal over time.

In response to the question about which task made them feel most accomplished, most participants chose the last task. This was also confirmed by a graph of valence and arousal for one of the participants, as shown in Figure 7. This could be due to the recency bias, or because the last task was a free-form task where the participants could implement a behaviour of their own choosing.

Figure 7 shows that the valence increased towards the end of the study. Peaks in the arousal value were more frequent in the later part of the session. In the free-form task 10, the participants were most interested in using emotive features of the robot—wagging the tail, closing eyes, and moving ears. This suggests that the participants preferred to program social behaviours in a robot.

The emotion recognition module worked well when the facial expression clearly indicated a strong emotion like happy, angry, or sad. However, with micro-expressions, the changes in valence and arousal were very small and could be considered as noise. It was observed that the participants’ faces were mostly neutral during the experiment, and their facial expressions barely changed regardless of whether they were doing well with the tasks or were facing difficulties.

## 7. Conclusions

The goal of this research was to study how affective feedback by an educational robot impacts learning outcomes; for example, in our particular case, does it lead to more interest in programming and computer science?

Our experimental data suggest that the Miro-E robot was perceived as friendly and likeable. However, we did not find a significant impact of the affective feedback of the robot on the participants—most of the differences can be attributed to other factors, such as programming experience or interest.

We did not find a strong positive or negative correlation of the robot’s behaviour to the participants’ responses. Previous studies in this area also report conflicting outcomes [7,9,45], suggesting that the link between social behaviour from robots and better outcomes for students is not so straightforward.

Our results show that the greatest affective outcome of the session was for students who had little experience or interest in programming before. This suggests that to maximise the impact of robot-assisted learning, it should be introduced early on. We also found that the affective expressions of the robot had a negative impact on its likeability.

### 7.1. Limitations

The sample size of nine participants was too small to draw statistical conclusions.

The feedback given by the robot was short and simple. This might have not been enough to generate observable effects, as most of the time the robot acted the same in both the experimental and the control groups.

As the study involved only one forty-minute session, the participants did not have much time to become confident with the robot’s programming interface.

Having a more uniform level of programming experience among the participants would have allowed creating tasks that were not too easy or too difficult for any participant, thereby eliminating one source of variations in the responses.

Participants were assigned to the experimental and the control groups before filling out the pre-test survey. Two participants with the most programming experience were placed in the experimental group. Due to the small sample size, the groups ended up with an unbalanced skill level and this difference had a visible effect on the answers.

### 7.2. Future Research

A study involving more participants over several sessions would answer the questions posed in this study with higher confidence. Future research could explore whether students who are learning programming with a robot would benefit more from the robot exhibiting social behaviours on its own, or from programming the robot to behave in social ways. Comparing a friendly-looking robot like Miro-E with a less inherently friendly-looking robot could give insight into how much the appearance of the robot influences its effectiveness as an educational tool.

Another issue for the future research is to study the potential drawbacks of affective feedback. It has been shown that too much emotional engagement from the robot can be a disadvantage and can lead to an increased cognitive load on the participant and worse outcomes [46]. Ethicists also argue that too much emotionality can lead to false relationships [47], or that the emotionality of robots is simply false [48]. These considerations must be taken into account when designing an affective educational robot.

## Figures and Tables

**Table 1:** Average responses to the survey.

Question	Total	Control	Experimental
Miro-E likeability	4.78	5	4.6
Session enjoyment	4.44	4.25	4.6
Programming experience	2.22	1.75	2.6
Interest in programming (pre-test)	3.77	3.75	3.8
Interest in programming (post-test)	4.11	4	4.2

## Data Availability

The experimental data is available by contacting the corresponding author.
